# Fatal Bluetongue Virus Serotype 3 Infection in Female Dogs: A Case Report from Alentejo, Portugal, 2024

**DOI:** 10.3390/v17020159

**Published:** 2025-01-24

**Authors:** Sílvia C. Barros, Diogo Maroco, Ana M. Henriques, Maria L. Costa, Alexandra Alves, Fernanda Ramos, Ana Duarte, Teresa Fagulha, Inês C. Varanda, Fábio Abade dos Santos, Ana C. Ferreira, Maria J. Barahona, Paulo M. Carvalho, Mariana Orvalho, Margarida D. Duarte

**Affiliations:** 1 Nacional Institute of Agrarian and Veterinarian Research, Quinta Do Marquês, Av. da República, 2780-157 Oeiras, Portugal; silvia.santosbarros@iniav.pt (S.C.B.); diogompmaroco@gmail.com (D.M.); margarida.henriques@iniav.pt (A.M.H.); fernanda.ramos@iniav.pt (F.R.); ana.duarte@iniav.pt (A.D.); teresa.fagulha@iniav.pt (T.F.); ines.caetano@iniav.pt (I.C.V.); fabio.abade@iniav.pt (F.A.d.S.); cristina.ferreira@iniav.pt (A.C.F.); maria.barahona@iniav.pt (M.J.B.); paulo.carvalho@iniav.pt (P.M.C.); 2 Faculdade de Ciências e Tecnologia, Universidade Nova de Lisboa, Campus da Caparica, 2829-516 Caparica, Portugal; 3 Hospital Veterinário Muralha de Évora, Rua Marechal Costa Gomes 9, 7005-145 Évora, Portugal; marialealcosta2000@gmail.com (M.L.C.); 2geral@hvetmuralha.pt (A.A.); geral@hvetmuralha.pt (M.O.); 4 Faculdade de Medicina Veterinária, Centre for Interdisciplinary Research in Animal Health (CIISA), Universidade de Lisboa, Avenida da Universidade Técnica, 1300-477 Lisboa, Portugal; 5 Associate Laboratory for Animal and Veterinary Sciences (AL4AnimalS), Avenida da Universidade de Lisboa, 1300-477 Lisboa, Portugal; 6 CECAV—Centro de Ciência Animal e Veterinária, Faculdade de Medicina, Veterinária de Lisboa, Universidade Lusófona, Centro Universitário de Lisboa, 1749-024 Lisboa, Portugal; 7 BioISI—Biosystems and Integrative Sciences Institute, Faculty of Sciences, University of Lisbon, 1749-016 Lisboa, Portugal; 8 I-MVET, Faculdade de Medicina, Veterinária de Lisboa, Universidade Lusófona, Centro Universitário de Lisboa, 1749-024 Lisboa, Portugal

**Keywords:** *Bluetongue virus*, BTV-3, serotype, dogs, abortion, infection, outbreak, transmission

## Abstract

The first official case of bluetongue virus serotype 3 (BTV-3) in Portugal was confirmed in sheep from the district of Évora in September 2024. Notably, mortality was observed in pregnant sheepdogs within the affected sheep flocks. This study presents four cases of pregnant dogs infected with BTV-3 in mid-September 2024, all of which aborted prior to death. BTV-3 was identified by RT-qPCR following initial positive results from pan-BTV RT-qPCR. The virus was subsequently isolated from the blood of one of the dogs in BHK-21 cells, and a partial sequence of the vp2 gene was obtained. This sequence showed 100% similarity to sheep BTV3/3234/PT2024, identified in Portugal in September 2024, as well as to BTV-3/NET2023, first reported in the Netherlands in 2023. These findings suggest that the viruses may be related or share a common origin. Co-infection with common canine viruses and pathogenic bacteria was ruled out, confirming that the fatalities were due to BTV-3 infection, probably by ingestion of sheep placenta after lambing. Our results confirm the potential for the transmission of BTV-3 to non-ruminant species, particularly carnivores, and, therefore, the wider ecological implications of this virus. In addition, the identification of transplacental transmission of BTV-3 in one of the dogs provides new evidence highlighting the complexity of the virus’ transmission mechanisms.

## 1. Introduction

*Bluetongue virus* (BTV) is a member of the genus *Orbivirus* of the family *Sedoreoviridae* [1]. It is a double-stranded RNA virus characterised by a 10-segmented genome, which allows genetic reassortment and the recurrent emergence of new strains. BTV is classified into more than 30 serotypes, with variations in virulence and pathogenicity influencing the epidemiology of the disease.

BTV is primarily transmitted by the bite of blood-feeding insects, particularly those of the genus *Culicoides*, which act as biological vectors [2,3]. These biting midges are widely distributed and play a crucial role in the life cycle of the virus, facilitating its spread among susceptible hosts. The virus is transmitted from infected to healthy animals during the viremic phase when the virus is present in the bloodstream of the host. The disease has a seasonal distribution, with increased transmission rates in the warmer months, which favour the proliferation of *Culicoides* midges.

Transmission by ingestion and transplacental transfer of BTV has been demonstrated, mainly for the BTV-8 [4,5,6,7]. The high abortion rate observed in sheep infected with BTV-3 suggests the possibility of vertical transmission of the virus.

BTV primarily affects wild [8,9,10] and domestic ruminants, including sheep [11], cattle, and goats [10], but there is increasing evidence that BTV can also infect non-ruminant species, including wild [12] and domestic carnivores [13,14,15,16,17,18,19]. Although these species may not act as primary reservoirs, cases of BTV infection in carnivores have been documented, often in association with their close proximity to infected livestock.

In September 2024, BTV-3 was detected in Portugal [20], with high genetic similarity to the newly identified BTV-3 strain reported in the Netherlands in 2023. Since then, official records have documented outbreaks caused by this serotype in ruminants, which remain the primary hosts of the virus, in several European countries. However, non-official reports have described the detection of BTV-3 in domestic dogs [21].

In this study, we report the first confirmed cases of BTV-3 detection in pregnant dogs in Portugal, associated with BTV-3 outbreaks in sheep. In addition, we present new evidence of transplacental transmission of BTV-3 in dogs.

## 2. Materials and Methods

### 2.1. Case Descriptions

All four animals involved in this study were animals living in rural environments with daily contact with sheep or cattle and corresponded to pregnant dogs that had aborted.

Common clinical findings included lethargy and tachypnoea, which progressed to severe systemic illness. Ultrasound examination revealed B-lines in the thoracic cavity, indicating possible pulmonary involvement. Vaginal discharge was present in all dogs, except for Dog 3, which had already undergone an abortion. In all cases, the foetuses were dead.

Biochemical analysis revealed elevated alkaline phosphatase and hyperbilirubinemia, consistent with cholestasis or liver dysfunction. In addition, hyponatremia, hyperphosphatemia, and uraemia suggested dehydration, electrolyte imbalance, and possible renal dysfunction (Table S1).

Case 1 was a six-year-old female German shepherd dog (hereafter referred to as Dog 1), approximately 30 days pregnant (Table S1), used to herd sheep that had ingested placental remains and aborted material from the flock. Initial clinical signs, which began on 19 September 2024, included lethargy, tachypnoea, and a small volume of brownish vaginal discharge, which increased over the following days. Despite these signs, other physical examination parameters remained within normal reference ranges. Blood tests revealed lymphopenia along with elevated levels of alkaline phosphatase, alanine aminotransferase, glucose, phosphate, and bilirubin. However, the levels of sodium, urea, calcium, and albumin were below the reference values (Table S1). Due to the lack of foetal viability and the deteriorating clinical condition of the dog, a surgical intervention was elected.

An ovariohysterectomy was performed, including the removal of the foetuses. Despite the surgery, the dog died two hours postoperatively on 20 September 2024.

Case 2 was a four-year-old female Rafeiro do Alentejo (hereafter referred to as Dog 2), approximately fifty days pregnant, with a vaccination history limited to rabies (Table S1). The dog had regular contact with the neighbouring flock of sheep. On 18 September 2024, the owners observed the dog consuming the carcass of a stillborn lamb. Clinical signs developed the following day, including lethargy, tachypnoea, and a small amount of brownish vaginal discharge, which worsened by 20 September. Despite these symptoms, other physical examination parameters were within normal limits. An ultrasound scan confirmed the absence of foetal heartbeats. Blood tests revealed lymphopenia, elevated levels of alkaline phosphatase, phosphatemia, bilirubinaemia, and uraemia, along with reduced natremia (Table S1). Medical treatment consisted of antibiotic therapy, the administration of antiemetics, and a gastric protector (Table S1). Due to the non-viable pregnancy and deteriorating clinical condition, an ovariohysterectomy was performed to remove the foetuses. However, the dog died five hours post-operatively, on 20 September 2024.

Case 3 was a two-year-old mixed-breed dog (hereafter referred to as Dog 3), which had been fully vaccinated. The dog had been in contact with cattle that had tested negative for the bluetongue virus. On 22 September 2024, the dog presented with clinical signs following the abortion of five incompletely developed foetuses at 50–60 days of gestation. Other signs included lethargy, bilateral muco-purulent ocular discharge, and tachypnoea, while other physical examination parameters were within normal limits. Blood tests revealed thrombocytopenia and monocytosis, with elevated levels of alkaline phosphatase, glucose, phosphate, bilirubin, and urea, and decreased levels of sodium and chloride levels (Table S1). Medical treatment included antibiotic therapy, along with the administration of analgesics, antiemetics, and a gastric protector (Table S1). The dog’s clinical condition deteriorated, and it died on 23 September 2024.

Case 4 was a 1-year-old vaccinated female Rafeiro do Alentejo dog, approximately 50 days pregnant (Table S1). The dog (hereafter referred to as Dog 4) had contact with other animals, including sheep.

The first clinical sign appeared on 21 September 2024, in the form of a small amount of brown vaginal discharge, indicating a possible abortion, as reported by the owners. On 23 September 2024, the dog was presented to the veterinary hospital with marked lethargy and a significant increase in brown vaginal discharge. An ultrasound revealed the absence of foetuses, heterogeneous echogenic content in the uterus, and thickened uterine walls, confirming an abortion. In addition, the kidneys exhibited hypoechoic cortices but retained corticomedullary differentiation, and the bladder was moderately distended with anechoic content. Blood tests showed thrombocytopenia, along with elevated levels of alkaline phosphatase, gamma-glutamyl transferase, phosphate, and bilirubin levels, and decreased albumin and sodium levels (Table S1). Between 25 September and 30 September 2024, the clinical condition worsened, with frequent episodes of vomiting, nausea, severe lethargy, abdominal distension, discomfort, hypothermia (temperatures ranging from 37.2 °C to 37.4 °C), and congested mucous membranes. Urine output was reduced (<0.5 mL/kg/h).

Subsequent blood analyses revealed markedly elevated urea, creatinine, phosphate, and potassium levels, as well as persistent hypoalbuminemia. Complete blood counts revealed leucocytosis and anaemia (Table S1). Medical treatment consisted of antibiotic therapy, together with the administration of non-steroidal anti-inflammatory drugs (NSAIDs), antiemetics, a gastric protector, an analgesic, and a diuretic (Table S1). Due to the unfavourable clinical course, the decision was made to euthanize the dog on 30 September 2024.

Importantly, no clinical signs of disease were observed in males and non-pregnant females living in the same environment as these four dogs.

### 2.2. Necropsy and Histopathology

Standard necropsies were performed on dogs 1, 2, and 3 according to established standard protocols. Tissue samples were collected from major organs, including the liver, lungs, heart, kidneys, spleen, gastrointestinal tract, reproductive organs, and foetuses and preserved at 4 °C for further analysis, including virological and bacteriological analysis. Histopathological analysis was performed on Dog 1 only. Tissue samples for histopathology were fixed in 10% neutral-buffered formalin for at least 24–48 h to ensure adequate preservation. After fixation, tissues were processed using a standard automated tissue processor and embedded in paraffin blocks. Sections of 4–5 µm thickness were cut from each block and mounted on glass slides. These sections were stained with haematoxylin and eosin (H&E) for general histopathological examination.

### 2.3. Nucleic Acid Extraction

Different organs were subjected to microbiological diagnosis (Table 1). Organs were mechanically homogenised in 20% (*w*/*v*) phosphate-buffered saline (PBS) with 0.5 mm zirconium beads (Sigma-Aldrich, St. Louis, MO, USA) using four cycles of 15 s at 3000 rpm with a 10 s interval (Precellys^®^ Evolution, Bertin Technologies, Montigny-le-Bretonneux, France), followed by clarification at 3000× *g* for 5 min. Total nucleic acids were extracted from 200 μL of the clarified supernatants, or from 200 μL of blood using the IndiMag^®^ Pathogen Kit (Indical, Leipzig, Germany) in a KingFisher Flex extractor (ThermoFisher Scientific, Waltham, MA, USA) according to the manufacturer’s protocol. Extractions were validated by 18S rDNA qPCR and RT-qPCR [22] for the detection of spiked synthetic RNA (VLP-RNA EXTRACTION, Meridian Life Science, Memphis, TN, USA).

### 2.4. Molecular Diagnosis

Bluetongue virus (BTV) was detected using the pan-BTV RT-qPCR system described by Hoffmann et al. (2008) [23]. Serotype-specific analysis for BTV-1, BTV-3, BTV-4, and BTV-8 was performed using the methods listed in Table 1.

The presence of various canine viral pathogens, including canine parvovirus, canine distemper virus, canine adenovirus, canine herpesvirus, and Aujeszky’s disease virus, as well as abortion-associated bacteria, in particular *Brucella* spp., *Chlamydia* spp., and *Coxiella* spp., was investigated using molecular methods, as detailed in Table 1.

### 2.5. Sequencing on the MinION™ Platform

For MinION sequencing, the supernatant of the third passage of BTV-3 infected BHK-21 cells was clarified by centrifugation at 3220× *g* for 10 min, filtered through a 0.22 µM membrane filter (Merck Millipore, Burlington, MA, USA), and treated with 10 U of TURBO DNase (Thermo Fisher Scientific, Waltham, MA, USA). Virus concentration was performed by ultracentrifugation at 91,500× *g* for 3 h. Viral RNA was extracted using the IndiMag^®^ Pathogen Kit (Indical, Leipzig, Germany) in a Kingfisher Flex extractor (Thermo Fisher Scientific, Waltham, MA, USA) according to the manufacturer’s protocol. Then, cDNA synthesis was carried out by adapting the SISPA protocol described by [31], using the SuperScript IV First-Strand Synthesis System kit (Thermo Fisher Scientific, Waltham, MA, USA). Extracted RNA was first annealed by preparing the following mixture: 5 μL of primer FR26RV-N (GCCGGAGCTCTGCAGATATCNNNNNN) [32] at 10 μM, 1 μL of dNTP mix (10 mM), 5 μL of RNA, and 2 μL of nuclease-free water. The reaction mixture was then incubated at 65 °C for 5 min before being briefly cooled on ice. Next, first strand synthesis was conducted by adding 4 μL of 5X RT Buffer, 1 μL of DTT (100 mM), 1 μL of SuperScript IV RT (200 U), and 1 μL of Ribonuclease Inhibitor (40 U). The final mixture was incubated at 23 °C for 10 min, followed by 50 °C for 45 min and 80 °C for 10 min, in a T100 Thermal Cycler (Bio-Rad Laboratories, Hercules, CA, USA). To remove any residual RNA molecules, 1 μL of E. coli RNase H (2 U) was added to the mixture, which was then incubated at 37 °C for 20 min. To complete the double-stranded form of the produced molecules, 1 μL (5 U) of DNA Polymerase I Large (Klenow) Fragment (New England Biolabs Inc., Ipswich, MA, USA), 2.5 μL of 10X NEBuffer 2 (New England Biolabs Inc., USA), and 0.5 μL of dNTP mix (10 mM) were added to 20 μL of cDNA. The final mixture was incubated at 25 °C for 15 min, followed by enzyme inactivation through the addition of 1 μL of EDTA (10 mM) and incubation at 75 °C for 20 min. The double-stranded DNA molecules were then amplified through PCR using the Speedy NZYTaq 2x Colourless Master Mix PCR kit (NZYtech, Lisbon, Portugal). The reaction mixture was prepared as follows: 25 μL of 2X Speedy NZYTaq 2× Colourless Master Mix, 5 μL of primer FR20RV (GCCGGAGCTCTGCAGATATC)*, and 15 μL of nuclease-free water. The amplification programme was one cycle of initial denaturation at 95 °C for 2 min, thirty cycles of amplification at 95 °C for 30 s, 55 °C for 20 s, 72 °C for 2 min, and a final extension step at 72 °C for 10 min. All PCR products were collected, visualised by agarose gel electrophoresis, and stored at −20 °C. Samples were purified, and libraries were prepared using the Rapid Barcoding Sequencing Kit (SQK-RBK114.24), following the manufacturer’s protocol (Oxford Nanopore Technologies, Oxford, UK). Sequencing was performed on a MinION Mk1B device with FLO-MIN114 flow cells for 3 h, employing a high-accuracy base-calling model with a minimum quality score (Q-score) threshold of 9. The resulting data were analysed and assembled using the Genome Detective Virus Tool (https://www.genomedetective.com).

A 1429 nt sequence from *vp2* gene was submitted to GenBank and attributed the accession number PQ654180.

### 2.6. Brucella spp. Isolation

Placenta, blood, and spleen samples were homogenised under sterile conditions in the minimum amount possible of sterile buffered saline (PBS, pH 6.8), and 0.2 mL of each tissue homogenate was seeded onto two plates of both Farrell’s selective media [33]. Plates were checked for growth after 5–10 days of incubation at 37 °C in both air and 10% CO_2_ atmospheres. A culture was considered positive if at least one *Brucella* colony-forming unit (CFU) was isolated. Suspect colonies were further identified and characterised by standard bacteriological procedures, as previously described by [34].

### 2.7. Viral Isolation

Blood samples collected in EDTA from dogs were processed according to the EURL protocol (Spain). Briefly, after three washes with phosphate-buffered saline (PBS) to remove plasma, erythrocyte lysis was performed by adding distilled water and incubating the samples on ice for 10 min. After centrifugation, the cell pellet was resuspended in PBS containing 1% antibiotic–antimycotic and incubated for 20 min at room temperature. The washed blood was used to inoculate Baby Hamster Kidney Fibroblast Cells (BHK-21). These cells were cultured in Glasgow Modified Eagle Medium (Gibco, Thermofisher, Waltham, MA, USA) supplemented with 5% foetal bovine serum (FBS). The monolayer was monitored daily for the appearance of cytopathic effects (CPE).

## 3. Results

### 3.1. Necropsy and Histopathology Data

**Figure 1:**
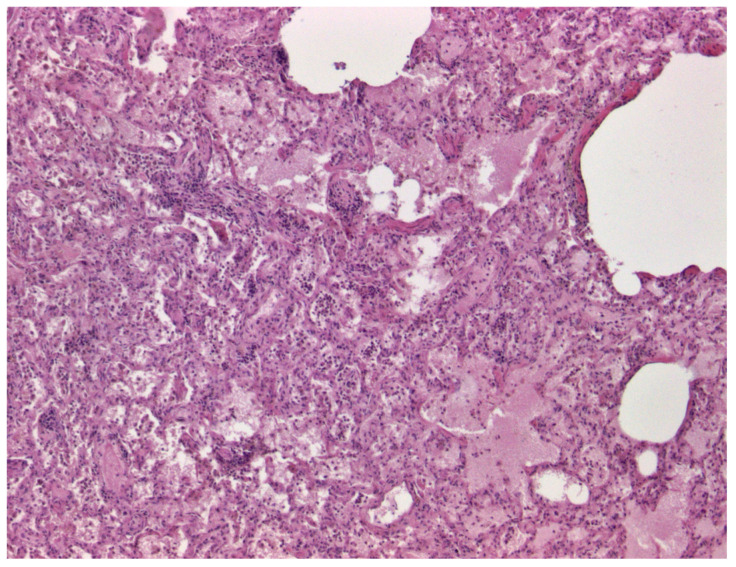
Lung histologic changes from Dog 1, H&E, 40×. Generalised alveolar oedema with mild to moderate infiltration of mononuclear inflammatory cells in the peribronchiolar region and alveolar spaces.

Necropsy findings in Dog 1 revealed congestion in several abdominal organs, including the intestinal tract, spleen, kidneys, and urinary bladder. The kidneys showed adhesions at decapsulation and a marked reduction in corticomedullary differentiation. The liver appeared haemorrhagic and decreased in consistency, while the lungs showed congestion and hepatisation of the right middle lobe. A pinkish frothy fluid was observed on the lung incision, consistent with alveolar oedema, further supporting the presence of significant pulmonary pathology. The lungs showed diffuse, generalised alveolar oedema that significantly impaired respiratory function. In addition, there was mild to moderate infiltration of mononuclear inflammatory cells in both the peribronchiolar region and the alveolar spaces (Figure 1). The spleen showed mild to moderate lymphoid depletion.

Necropsy findings in Dog 2 revealed pulmonary congestion and hepatisation of one lung lobe, with pinkish frothy fluid suggestive of alveolar oedema seen on the lung incision. In the abdominal cavity, the liver showed a congested appearance with reduced consistency, while the kidneys showed marked congestion.

Necropsy of Dog 3 showed a haemorrhagic liver with altered consistency, while the kidneys were markedly congested. The uterus contained brownish, foul-smelling material. Pulmonary findings included congestion and hepatisation of the right middle lobe, and an incision of the lungs revealed pinkish frothy fluid consistent with alveolar oedema.

### 3.2. Microbiological Findings

BTV was detected in the tissues of the four dogs using the pan-BTV RT-qPCR system [23], indicating the presence of BTV-RNA. Serotype-specific analysis, using the methods listed in Table 1, confirmed BTV-3 in the blood samples of dogs 2, 3, and 4, and in the liver, spleen, lungs, and kidney from dogs 1 and 3. In addition, the spleen of Dog 2 tested positive for BTV-3. Of note, both the placenta and foetus of Dog 2 and the uterus of Dog 3, showed positive Ct values, with the lowest value observed in the placenta (Ct 16.33). The Ct values obtained in the different tissue matrices are shown in Table 2.

None of the samples tested positive for other serotypes, namely BTV-1, BTV-4, and BTV-8. The results showed negative for all the other viruses and bacteria tested. Laboratory testing was not conducted on dogs that appeared to be healthy.

### 3.3. Virus Isolation

Virus isolation in BHK-21 cells was successful for Dog 2. The characteristic CPE, i.e., rounding and ballooning of the cells followed by detachment from the surface of the flask, was evident at 72 h after infection in the first passage. The isolation was confirmed by RT-qPCR of the cell supernatants, which gave a Ct of 15.0 and 11.41 in the first and second passage, respectively. Isolate was named BTV3/19767/PT2024.

#### Molecular Characterisation of the Viral Isolate

Sequencing of the partial *vp2* gene (PQ654180) revealed 100% similarity to the sheep BTV3/3234/PT2024 (PQ609286, PQ609287), identified in Portugal in September 2024, indicating that the virus circulating in cattle and sheep can be the same strain affecting dogs. Additionally, the similarity with the homologous region of BTV-3/NET2023 (OR603993.1) was also 100%, suggesting a potential common origin for these viruses.

## 4. Discussion

Epidemiological data indicate that the four affected dogs were not from the same farm but were located within the same geographic region of Alentejo, specifically in the district where BTV-3 was first detected in Portugal [20]. Although no specific timeline is available for the outbreaks in infected livestock across the four farms, the dogs were housed on separate properties and did not come into direct contact with each other. However, one of the infected dogs (Dog 2) had contact with a non-pregnant dog that showed no clinical signs. There is no clear information on whether Dog 2 co-habitant also became infected through ingestion of infected material or by a *Culicoides* bite. If the infection did occur via one of these routes, it appears this female developed a subclinical course of the disease, as no clinical signs were observed. This may suggest that non-pregnant dogs may experience a subclinical progression of the infection, highlighting a strong association between pregnancy and severe reproductive clinical disease with fatal outcomes following BTV-3 infection. However, it cannot be ruled out that transmission dynamics may differ across dogs exposed to the virus independently of its reproductive stage. Importantly, aside from the four pregnant females reported in this study, no other females were pregnant at the time on the farms, and no additional clinical cases were observed in all the other dogs on the premises. Testing asymptomatic non-pregnant females and males would have provided valuable insights into the potential spread of the infection within the canine population in the area, helping to better define the full scope of transmission.

Although BTV was thought to be transmitted to dogs through *Culicoides* biting midges [19], BTV infection in carnivores appears to occur primarily through the ingestion of BTV-infected material, such as sheep placenta and stillbirths. Abortion and death have been observed following infection. Available reports refer to BTV-8 and BTV-4 in lynx [14,18] and BTV-11 in dogs [15], while some reports do not specify the serotype involved [16,17].

In ruminants, oral transmission of BTV-8 and BTV-11 has also been demonstrated by ingestion of colostrum and infected placenta [4,7].

In addition to vector-borne and oral transmission, several alternative routes of BTV transmission have been proposed. These include: (i) venereal transmission via infected semen [35,36], (ii) transplacental transmission leading to in utero infection [6,7,37,38,39,40], (iii) mechanical transmission by insect vectors [41], and (iv) transmission by direct contact [42].

Only one case of canine infection with BTV-3 has been reported before, published online as a press release by Wageningen University on 20 December 2023 and by the Western Canadian Animal Health Network. The 3.5-year-old pregnant dog lived on a Dutch dairy farm with cattle and sheep and was infected with BTV-3/NET2023, by ingestion of sheep afterbirth or by midge bite.

In our reported cases, two female dogs are known to have ingested sheep afterbirth and aborted material, behaviours that could have facilitated cross-species transmission of the virus. However, it cannot be excluded that the other dogs which did not develop signs of disease, may have also ingested infected material.

The reproductive effects of BTV-3 infection in pregnant dogs, particularly its potential role in inducing abortion and subsequent mortality, remain poorly understood. If BTV-3 shares pathophysiological mechanisms with BTV-8, it is plausible that infection may lead to placentitis, foetal distress, and impaired uterine function, culminating in abortion.

The hypothesis of oral transmission is supported by the documented case of BTV-8 infection in two Eurasian lynxes (*Lynx lynx*) in a zoo in Belgium. These lynxes were fed ruminant foetuses and stillbirths from nearby farms during a BTV-8 outbreak, suggesting a possible oral route of transmission [18]. In addition, previous studies have suggested that seroconversion to BTV can occur in carnivorous species as a result of oral exposure [12]. Taken together, these findings increase the likelihood of inter-species transmission of BTV-3 via the oral route and highlight the need for further investigation into the dynamics of cross-species transmission.

An interesting finding of our study was the detection of BTV-3 in a dog foetus. While there is currently limited information on the potential for natural transplacental transmission of BTV-3, evidence for such transmission has been documented in goats with BTV-8 [5]. Since the initial detection of BTV-3 in September 2024, we have consistently identified viral RNA of BTV-3 in the foetuses and placentas of cattle, goats, and sheep diagnosed as BTV-3 positive, further supporting the evidence of vertical transmission of this virus.

Although BTV-11 has already been detected in a dog foetus [15], to our knowledge this is the first identification of BTV-3 in the canine foetus, supporting transplacental transmission of this serotype in dogs. This observation highlights the need for further investigation to clarify the mechanisms of BTV-3 transmission and to assess the implications for canine health in regions where BTV-3 is endemic.

Necropsy and histopathological findings from Dog 2 revealed mild to moderate infiltration of mononuclear inflammatory cells in the peribronchiolar region and alveolar spaces, along with diffuse and generalised alveolar oedema. This oedema compromised respiratory function, ultimately resulting in death. To our knowledge, these histopathological findings are the first to be reported in association with BTV-3 infection in dogs.

The cross-species transmission reported here highlights the need to further investigate the impact of BTV on non-ruminant populations, particularly in rural areas where domestic and wild carnivores frequently interact with livestock. As the epidemiology of BTV continues to evolve, it is essential to monitor its impact on all susceptible species to develop effective control measures and protect the health of domestic and wild animals.

## Figures and Tables

**Table 1:** Pathogens investigated in this study by molecular methods.

Viruses	Kit Used	Genomic Region Targeted	Size of Amplicon	Tissue Used	Reference of the Method
Bluetongue virus	One-step NZYSpeedy RT-qPCR Probe kit	*NS3* gene	97 bp	blood	[23]
BTV-1	One-step NZYSpeedy RT-qPCR Probe kit	*vp2* gene	194 bp	blood	In house, unpublished
BTV-3	One-step NZYSpeedy RT-qPCR Probe kit	*vp2* gene	74 bp	blood, foetus, intestine, kidney, liver, lung, spleen, uterus,	[24]
BTV-4	One-step NZYSpeedy RT-qPCR Probe kit	*vp2* gene	109 bp	blood	In house, unpublished
BTV-8	One-step NZYSpeedy RT-qPCR Probe kit	*vp2* gene	66 bp	blood	WOAH recommended method
Canine parvovirus	NZYSpeedy qPCR Probe Master Mix (2x)	*vp2* gene	93 bp	kidney, liver, lung, spleen	[25]
Canine distemper virus	One-step NZYSpeedy RT-qPCR Probe kit	*N* gene	161 bp	blood, kidney, liver, lung, spleen	In house, unpublished
Canine adenovirus	NZYSpeedy qPCR Probe Master Mix (2x)	*hexon* gene	80 bp	blood, spleen, lung, liver, kidney	[26]
Canine herpesvirus	NZYSpeedy qPCR Probe Master Mix (2x)	*gB* gene	136 bp	blood, spleen, lung, liver, kidney	[27]
Aujeszky’s disease virus	NZYSpeedy qPCR Probe Master Mix (2x)	*gB*	96 bp	blood, spleen, liver, lung, kidney	[28]
		*gE*	136 bp		
*Brucella* spp.	TaqMan Universal PCR Master Mix	*bcsp31*	224 bp	blood, spleen, placenta	[29]
*Chlamydia* spp.	GoTaq^®^ G2 DNA Polymerase (Promega, Madison, WI, USA)	*16S rDNA*	270 pb	placenta	[30]
*Coxiella* spp.	AdiaLyo Coxiella (BioX Diagnostics, Rochefort, Belgium)	*IS1111*	undisclosed	placenta	commercial ADL14Y1-100-ADIALYO kit

**Table 2:** Ct values obtained in the biological samples from Dog 1, 2, 3, and 4, using the BTV-3 RT-qPCR [24].

Dog ID	Biological Matrix	Ct Value
1	liver	20.86
	spleen	18.74
	lung	23.27
	kidney	22.32
2	blood	17.40
	spleen	18.59
	foetus (liver and heart)	27.57
	placenta	16.33
3	blood	23.93
	uterus	28.26
	liver	22.12
	spleen	22.05
	lung	25.15
	kidney	24.72
4	blood	25.28

## Data Availability

The data supporting the results of this study can be obtained by contacting the authors; however, farm identities and dogs’ names are not disclosed due to confidentiality and the right to privacy of the property owners. A nucleotide sequence has been submitted to GenBank under the accession number PQ654180.

## Supplementary Materials

The following supporting information can be downloaded at: https://www.mdpi.com/article/10.3390/v17020159/s1, Table S1. Clinical data from Dogs included in this study.
