# Supplementary material for: Fatal Bluetongue Virus Serotype 3 Infection in Female Dogs: A Case Report from Alentejo, Portugal, 2024
Source: Viruses. 2025 Jan 24;17(2):159. doi: 10.3390/v17020159 (PMC11860487; doi:10.3390/v17020159)
Supplement: Supplementary file 1 [file viruses-17-00159-s001.zip › viruses-3318544-supplementary.pdf]

Table S1. Clinical data from Dogs included in this study.

	Dog 1	Dog 2	Dog 3	Dog 4	
Age (years)	6	4.5	2.5	1	
Vaccination History	Rabbies, CPV [1], CDV [2], Lept [3], CPIV [4], CAV-2 [5]	Rabbies	Rabbies, CPV [1], CDV [2], Lept [3], CPIV [4], CAV-2 [5]	Rabbies, CPV [1], CDV [2], Lept [3], CPIV [4], CAV-2 [5]	
Clinical signs	lethargy, tachypnoea, vaginal discharge, death of foetus	lethargy, tachypnoea, vaginal discharge, death of foetus	abortion, lethargy, tachypnoea death of foetus	vaginal discharge, tachypnoea, death of foetus	
Time of pregnancy (days)	30	50	50–60	50	
Biochemistry & Haematology					**Reference values**
Haematocrit				21.8–31.7%↓	39–56%
Leucocytes				18–30 × 10^9[6]↑	6–17 × 10^9
Lymphocytes	6↓	9.3↓			12–30%
Platelets	-	-	72 × 10^9↓	46 × 10^9↓	117–460 × 10^9
Monocytes	-	-	10.9↑	-	2–9%
ALP	229↑	89↑	251↑	115↑	13–83 UL/L
ALT	458↑	-	-	-	17–78 U/L
Glycaemia	136↑	132↑	255↑	-	75–128 mg/dL
Bilirubin	1.1↑	0.6↑	1.1↑	1.9↑	0.1–0.51 mg/dL
Cl^-	-	-	96↓	-	102–117 mEq/L
Na^+	137↓	133↓	125↓	135↓	141–152 mEq/L
K^+	9.0↓	-	-	5.1[6]↓	9.3–12.1 mg/dL
P	13.2↑	5.5↑	5.5↑	13.6/15[6]↑	1.9–5 mg/dL
Urea	68.8↑	39.0↑	86.4↑	140[6]↑	9.2–29.2 mg/dL
Creatinine	-	-	-	14.01[6]↑	0.4–1.4
Albumin	2.1↓	-	-	2.5/2.3[6]	2.6–4 mg/dL
Treatment [7]	-				

Antibiotic		Cefazolin<br>Enrofloxacine	Cefazolin	Ampicilin<br>Enrofloxacine<br>Metronidazol
Proton-pump inhibitor		Omeprazole	Omeprazole	Omeprazole
Antiemetic		Maropitant	Maropitant	Maropitant
Analgesic			Methadone	Methadone
NSAI				Meloxicam
Diuretic				Furosemide
Euthanized	no	no	no	yes

1. CPV-canine parvovirus; 2. CDV-canine distemper virus; 3. Lept-Leptospira; 4. CPIV-canine parainfluenza virus; 5. CAV-canine adenovirus; 6. values obtained 6 days after the remaining values; 7. All medication was administered at the recommended dose intravenously except for Meloxicam, which was administered subcutaneously.
